# Microbiota analysis and transient elastography reveal new extra-hepatic components of liver steatosis and fibrosis in obese patients

**DOI:** 10.1038/s41598-020-79718-9

**Published:** 2021-01-12

**Authors:** Nicolas Lanthier, Julie Rodriguez, Maxime Nachit, Sophie Hiel, Pierre Trefois, Audrey M. Neyrinck, Patrice D. Cani, Laure B. Bindels, Jean-Paul Thissen, Nathalie M. Delzenne

**Affiliations:** 1 Laboratory of Hepatology and Gastroenterology, Institut de Recherche Expérimentale et Clinique, UCLouvain, Université catholique de Louvain, Brussels, Belgium; 2 Service d’Hépato-gastroentérologie, Cliniques universitaires Saint-Luc, UCLouvain, Brussels, Belgium; 3 Metabolism and Nutrition Research Group, Louvain Drug Research Institute, UCLouvain, Université catholique de Louvain, Avenue E. Mounier, box B1.73.11, 1200 Brussels, Belgium; 4 Medical and Imaging Department, Cliniques universitaires Saint-Luc, UCLouvain, Brussels, Belgium; 5 WELBIO - Walloon Excellence in Life Sciences and BIOtechnology, UCLouvain, Université catholique de Louvain, Brussels, Belgium; 6 Pole of Endocrinology, Diabetes and Nutrition, Institut de Recherche Expérimentale et Clinique, UCLouvain, Université catholique de Louvain, Brussels, Belgium; 7 Service d’Endocrinologie, diabétologie et nutrition, Cliniques universitaires Saint-Luc, UCLouvain, Brussels, Belgium

**Keywords:** Microbiology, Physiology, Biomarkers, Gastroenterology, Risk factors

## Abstract

Obesity could lead to metabolic dysfunction-associated fatty liver disease (MAFLD), which severity could be linked to muscle and gut microbiota disturbances. Our prospective study enrolled 52 obese patients whose MAFLD severity was estimated by transient elastography. Patients with severe steatosis (n = 36) had higher ALAT values, fasting blood glucose levels as well as higher visceral adipose tissue area and skeletal muscle index evaluated by computed tomography. Patients with fibrosis (n = 13) had higher ASAT values, increased whole muscle area and lower skeletal muscle density index. In a multivariate logistic regression analysis, myosteatosis was the strongest factor associated with fibrosis. Illumina sequencing of 16S rRNA gene amplicon was performed on fecal samples. The relative abundance of fecal *Clostridium sensu stricto* was significantly decreased with the presence of liver fibrosis and was negatively associated with liver stiffness measurement and myosteatosis. In addition, 19 amplicon sequence variants were regulated according to the severity of the disease. Linear discriminant analysis effect size (LEfSe) also highlighted discriminant microbes in patients with fibrosis, such as an enrichment of Enterobacteriaceae and *Escherichia/Shigella* compared to patients with severe steatosis without fibrosis. All those data suggest a gut-liver-muscle axis in the pathogenesis of MAFLD complications.

## Introduction

The prevalence of obesity has reached epidemic proportions worldwide. Epidemiological studies reveal that obesity is associated to complications such as diabetes mellitus, cardiovascular and kidney diseases, cancers and musculoskeletal disorders^1^.

Obesity is also associated with liver steatosis development^2,3^. Several studies have pointed the critical role of the adipose tissue, and in particular its visceral location, in obesity-related metabolic disorders^4^. More recently, the presence of low muscle mass and/or high muscle fat infiltration (often referred to as “myosteatosis”) has been evidenced as another feature of diabetes^5,6^ and metabolic dysfunction-associated fatty liver disease (MAFLD)^7,8^. In cirrhosis, muscles alterations (*i.e.* myosteatosis and sarcopenia) are strong and independent predictors of mortality^9^. Interestingly, obesity, MAFLD and diabetes are all characterized by alterations of the composition of the gut microbiota, also referred as dysbiosis^10^, while there is no consensus on a precise bacterial signature related to the severity of those diseases^11^. Interestingly, previous evidences in rodent models indicated the causal role of the gut microbiota in MAFLD development. Indeed, the fecal microbiota transplantation (FMT) from patients with non-alcoholic steatohepatitis to germ-free mice exacerbated the metabolic features (including hepatic steatosis) in high-fat-diet fed-and inoculated mice^12^. FMT from obese women with hepatic steatosis into recipient mice also resulted in a rapid accumulation of hepatic triglycerides^10^. On this basis, several human studies compared the gut microbiota composition between patients with MAFLD and healthy subjects in order to identify a microbial signature associated with liver complications (for review^13^). Recently, Lelouvier et al*.* demonstrated that the fecal microbiota was impacted in patients with liver fibrosis and showed that some changes in the blood bacterial DNA sequences are associated with fibrosis in obese individuals^14^. Human studies have just started to describe microbiome signatures in MAFLD, but deciphering the bacterial signature associated with liver alterations, and more specifically associated with the severity of the disease, would be interesting as new indicators for MAFLD pathogenesis.

MAFLD can be evaluated through the development of non-invasive techniques^3,15^**.** Transient elastography (TE) (FIBROSCAN) is able to evaluate liver steatosis and fibrosis, respectively through the generation of a controlled attenuation parameter (CAP) and a liver stiffness measurement (LSM)^16,17^. Adapted cut-offs have been proposed for MAFLD patients due to steatosis influence on LSM^18^. TE is now widely recognized as one of the best and adequate screening tool for liver fibrosis in MAFLD^3,15^**,** with available quality criteria^19^.

In our study, we have evaluated the feasibility of both CAP and LSM measurement using transient elastography (TE) (with M or XL probes) to analyze the spectrum of MAFLD severity (*i.e.* steatosis and fibrosis degree) in obese individuals recruited prospectively in St Luc Hospital (Belgium) in the Food4Gut study^20^. This allowed us to elaborate the link between MAFLD severity and extra-hepatic alterations incriminating adiposity, skeletal muscle dysfunction and the gut microbiome.

## Results

### Patient’s population

Fifty-two patients (26 males and 26 females, mean age 50 years) were recruited from January 2016 to May 2018 and included in the analysis. All patients were obese, with a mean BMI of 36 kg/m^2^. Mean abdominal perimeter was 116 cm and 15 subjects were treated for hyperglycemia in a context of type 2 diabetes. The profile of liver enzyme was typical of MAFLD with a mean small increase predominantly in ALAT, and slight increase in ASAT and γGT. This detailed data is shown in Table 1.

**Table 1:** Patients population.

Gender	N (M/F)	26/26
Age	years, mean (min–max)	50 (21–65)
Body weight	kg, mean (min–max)	105 (74–162)
Body mass index	kg/m^2^, mean (min–max)	36 (30–56)
Waist	cm, mean (min–max)	116 (95–168)
Antidiabetic drug	N (%)	15 (29)
Antihypertensive treatment	N (%)	35 (67)
ALAT	UI/L, mean (min–max)	41 (8–130)
ASAT	UI/L, mean (min–max)	27 (10–67)
γGT	UI/L, mean (min–max)	47 (9–144)
Fasting triglyceride	mg/dL, mean (min–max)	153 (53–328)
Fasting blood glucose	mg/dL, mean (min–max)	105 (80–176)

*ASAT* aspartate aminotransferase, *ALAT* alanine aminotransferase, *γGT* γ-glutamyl transferase.

### Liver stiffness and controlled attenuation parameter measurement

Reliable LSM were obtained in 49 patients (94%) and included in the subsequent analysis (Table 2). Indeed, we had one failure in LSM (no valid measurement due to an important liver cyst). Two examinations gave unreliable results, one for a rate of valid measurement below 70% and the other for an IQR/median higher than 30%. The M probe was used in 30 patients (61%) and the XL probe in 19 patients (39%) (Table 2). Mean liver stiffness was low at 6.5 kPa (mean IQR: 1.2). Thirteen patients (27%) had liver stiffness values suggestive of fibrosis (Table 2). CAP measurement was compatible with liver steatosis in all patients. Mean CAP value was 325 dB/m (mean IQR: 30). The majority of the patients (n = 36, 73%) had severe (grade S3) steatosis (Table 2). Thirteen patients (27%) had non-severe steatosis, either slight (grade S1, n = 3) or moderate (grade S2, n = 10) steatosis. Hence, both M and XL probes had high success rate in this obese population and MAFLD was found in all patients, among whom a quarter presented high liver stiffness compatible with fibrosis.

**Table 2:** Transient elastography results.

Failure/unreliable results	N (%)	3 (6)
M probe	N (%)	30 (61)
XL probe	N (%)	19 (39)
Mean liver stiffness	kPa ± SD	6.5 ± 3.2
Mean liver stiffness M probe	kPa ± SD	6.6 ± 3.3
Mean liver stiffness XL probe	kPa ± SD	6.4 ± 3.4
Fibrosis (LSM ≥ 7.8 kPa with M probe, ≥ 6.4 kPa with XL probe)	N (%)	13 (27)
Mean CAP value	dB/m ± SD	325 ± 45
Severe steatosis (CAP ≥ 296 dB/m)	N (%)	36 (73)

*LSM* liver stiffness measurement, *CAP* controlled attenuation parameter.

### Indicators of steatosis severity

**Figure 1:**
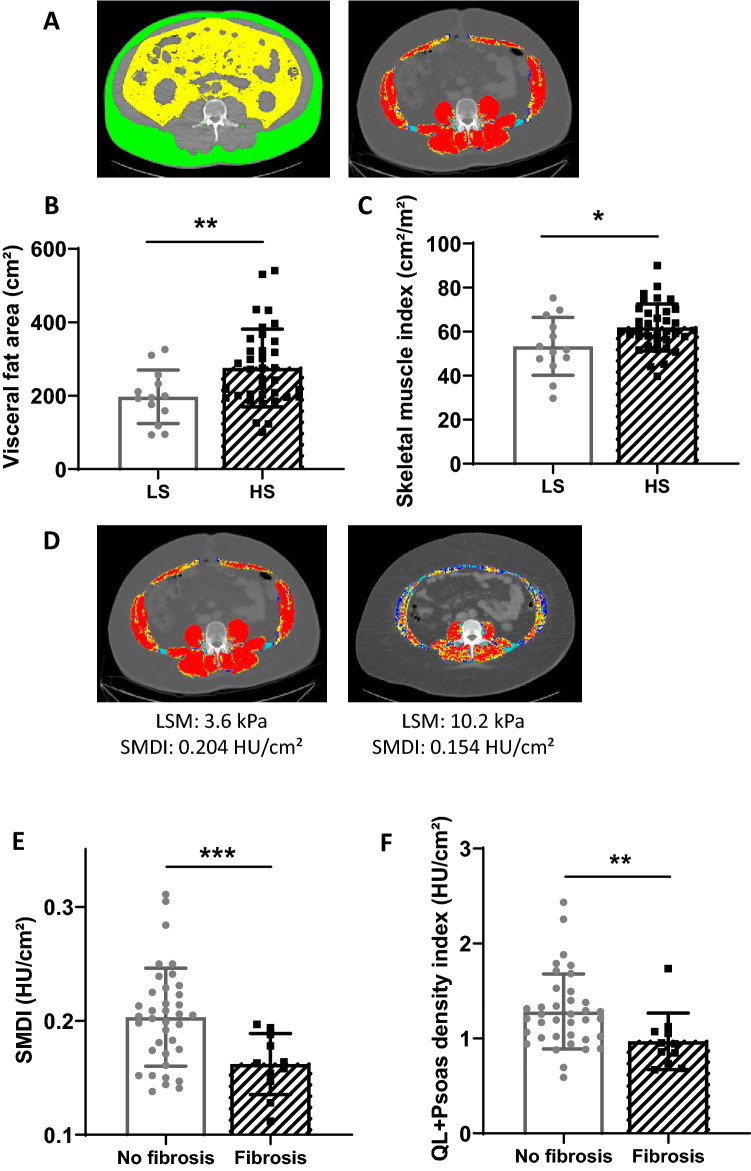
Body composition evaluation using computed tomography (CT) scan depending on the steatosis and fibrosis grades. Representative picture of the subcutaneous adipose tissue and the visceral fat are evidenced in yellow and green respectively at the third lumbar level, while the muscle area appears in red in patients (**A**). Mean visceral fat area (**B**) and skeletal muscle index (**C**) depending on the steatosis grade. n = 13 for patients with slight/moderate steatosis (LS), n = 36 for patients with severe steatosis (HS). Representative comparison of two MAFLD patients with obesity (**D**): patient at the left had normal liver stiffness measurement (LSM) with XL probe and normal mean muscle attenuation (skeletal muscles in red) and normal skeletal muscle density index (SMDI), whereas the patient at the right had a high LSM (with the XL probe also compatible with fibrosis), low mean muscle attenuation (skeletal muscles in red, yellow and blue) and low SMDI. Whole muscle density index (**E**) in the two groups (no fibrosis versus fibrosis) and quadratum lomborum (QL) + psoas density index (**F**) (n = 36 for patients with no fibrosis, n = 12 for patients with fibrosis. HU: Hounsfield unit. *p < 0.05, **p < 0.01, ***p < 0.001. CT-scan images were processed with the Slice-O-Matic software, version 4.3 (Tomovision, Montreal, Canada). Heatmap representation was performed using Graphpad Prism software version 8 (www.graphpad.com).

Compared to patients with low steatosis (LS, grade S1 or grade S2), patients with severe steatosis (HS, grade S3) were similar in terms of age, body weight and BMI (Table 3). A significantly higher proportion of men had severe steatosis. Liver tests revealed significantly higher values of ASAT (28.6 vs. 21.0 UI/L) and predominantly ALAT (45.3 vs. 28.1 UI/L) in patients with severe steatosis. Mean fasting blood glucose was also significantly higher (Table 3). However, the number of patients taking a medication for diabetes was the same in the two groups (23% for patients with non-severe steatosis vs 30% for patients with severe steatosis). Regarding body composition, resistance, fat free mass and total body fat were the same in the two groups. Subcutaneous fat area, evaluated by CT-scan, was also the same in the two groups. However, CT-scan (Fig. 1A) revealed significant differences between the two groups, patients with severe steatosis being characterized by a significantly higher visceral fat area (Fig. 1B) and skeletal muscle index (SMI) (Fig. 1C) compared with patients with non-severe steatosis. Multivariate logistic regression analysis showed that SMI was the best independent indicator of severe steatosis in obese patients (Table 3). Hence, increased liver steatosis is associated with a higher, rather than a lower skeletal muscle mass in obese patients. To evaluate myosteatosis, we computed a skeletal muscle density index (SMDI), as described in the methods. Indeed, muscle density (Fig. 1D) is a surrogate for lipid concentration and muscle area is a surrogate for muscle volume. Scaling muscle density by muscle area reflects on the absolute lipid quantity within skeletal muscle. Muscle composition evaluated by whole SMDI was not statistically different between the two groups (Table 3).

**Table 3:** Analysis of patients with severe steatosis.

	Low steatosis (LS) N = 13	Severe steatosis (HS) N = 36	*p* value	*p* value Logistic regression analysis
**Clinical parameters**				
Age (years)	49.2	50.1	0.79	
Weight (kg)	98.1	108.1	0.06	
BMI (kg/m^2^)	34.3	36.5	0.16	
Waist (cm)	112.0	116.9	0.18	
Waist to hip ratio	0.95	0.980	0.07	
Sex (M/F)	3/10	22/14	**0.02**	0.231
Antidiabetic drug (n/total)	3/13	11/36	0.61	
**Biological results**				
ASAT (UI/L)	21.0	28.6	**0.02**	
ALAT (UI/L)	28.1	45.3	**0.006**	0.068
γGT (UI/L)	40.0	50.6	0.29	
Fasting glycemia (mg/dL)	95.1	109.2	**0.01**	0.091
**Bioimpedance analysis**				
Resistance	480.5	440.1	0.15	
Fat free mass (kg)	62.4	68.4	0.19	
Total body fat (kg)	37.9	39.6	0.68	
**CT-scan data**				
Subcutaneous fat area (cm^2^)	340.4	363.1	0.62	
Visceral fat area (cm^2^)	197.3	275.4	**0.007**	0.567
Whole muscle area (cm^2^)	155.4	182.4	0.07	
Skeletal muscle index (cm^2^/m^2^)	53.3	61.9	**0.04**	**0.040**
Skeletal muscle density (HU)	32.2	33.1	0.67	
Skeletal muscle density index (HU/cm^2^)	0.216	0.186	0.06	

Results are given as mean value (and compared with a bilateral Student-t-test). Number of patients are compared with a chi-squared test. All significant parameters from the univariate analysis (except for collinear values) included in the logistic regression analysis are reported in the last column.

*ASAT* aspartate aminotransferase, *ALAT* alanine aminotransferase, *BMI* body mass index, *γGT* γ-glutamyl transferase, *HU* Hounsfield unit.

Significant differences are presented in bold.

### Independent predictors of fibrosis

We determined factors associated with fibrosis in this population (i.e. LSM ≥ 7.8 kPa with M probe or ≥ 6.4 with XL probe). Patients with fibrosis were predominantly males (Table 4). Body weight and waist circumference were significantly higher compared with patients without fibrosis; however, BMI was not significantly increased (Table 4). Except for ASAT, patients with or without fibrosis had similar clinical biology profiling. In particular, fasting glycaemia levels were the same in the two groups (Table 4). Intriguingly, patients with fibrosis were characterized by a significantly higher fat free mass at bioimpedance (BIA) analysis (Table 4). CT-scan data revealed similar pattern, showing a non-significant higher amount of fat area (either subcutaneous or visceral) but a significantly increased whole muscle area (199.6 vs. 165.8 cm^2^) in patients with fibrosis (Table 4). However, the skeletal muscle area scaled on height squared (yielding the SMI) was no longer significantly different between both groups (Table 4). Interestingly, the whole SMDI, reflecting “absolute” myosteatosis, was significantly lower in patients with fibrosis (0.160 vs. 0.202 HU/cm^2^) (Table 4 and Fig. 1E). Of note, SMDI remained significantly lower in patients with fibrosis when only posterior muscles (i.e. psoas, quadratum lumborum and erector spinae) were considered (0.98 vs. 1.28 HU/cm^2^) (Fig. 1F). After multivariate logistic regression analysis, the SMDI remained the only significant factor associated with fibrosis (Table 4). Hence, patients with fibrosis have a higher degree of muscle fat infiltration than those without fibrosis.

**Table 4:** Key characteristics of liver fibrosis.

	No fibrosis N = 36	Fibrosis N = 13	p-value	p-value Logistic regression analysis
**Clinical parameters**				
Age (years)	49.7	50.3	0.89	
Weight (kg)	100.8	118.3	**0.003**	
BMI (kg/m^2^)	34.9	38.6	0.14	
Waist (cm)	112.6	124.0	**0.02**	0.447
Waist to hip ratio	0.96	1.01	0.08	
Sex (M/F)	14/22	11/2	**0.005**	0.436
Antidiabetic drug (n/total)	8/36	6/13	0.10	
**Biological results**				
ASAT (UI/L)	23.8	34.2	**0.04**	0.111
ALAT (UI/L)	35.7	54.6	0.08	
γGT (UI/L)	46.1	52.8	0.52	
Fasting glycemia (mg/dL)	105.2	107.0	0.81	
**Bioimpedance analysis**				
Resistance	447.2	421.5	0.29	
Fat free mass (kg)	63.5	76.2	**0.002**	
Total body fat (kg)	38.1	42.1	0.33	
**CT scan data**				
Subcutaneous fat area (cm^2^)	337.5	411.3	0.23	
Visceral fat area (cm^2^)	237.6	302.0	0.07	
Whole muscle area (cm^2^)	165.8	199.6	**0.009**	
Skeletal muscle index (cm^2^/m^2^)	57.7	64.2	0.10	
Skeletal muscle density (HU)	32.6	31.8	0.72	
Skeletal muscle density index (HU/cm^2^)	0.202	0.160	**0.0004**	**0.014**

Results are given as mean value (and compared with a bilateral Student-t-test). Number of patients are compared with a chi-squared test. All significant parameters from the univariate analysis (except for collinear values) included in the logistic regression analysis are reported in the last column.

*ASAT* aspartate aminotransferase, *ALAT* alanine aminotransferase, *BMI* body mass index, *γGT* γ-glutamyl transferase, *HU* Hounsfield unit.

Significant differences are presented in bold.

### Gut microbiota changes

**Figure 2:**
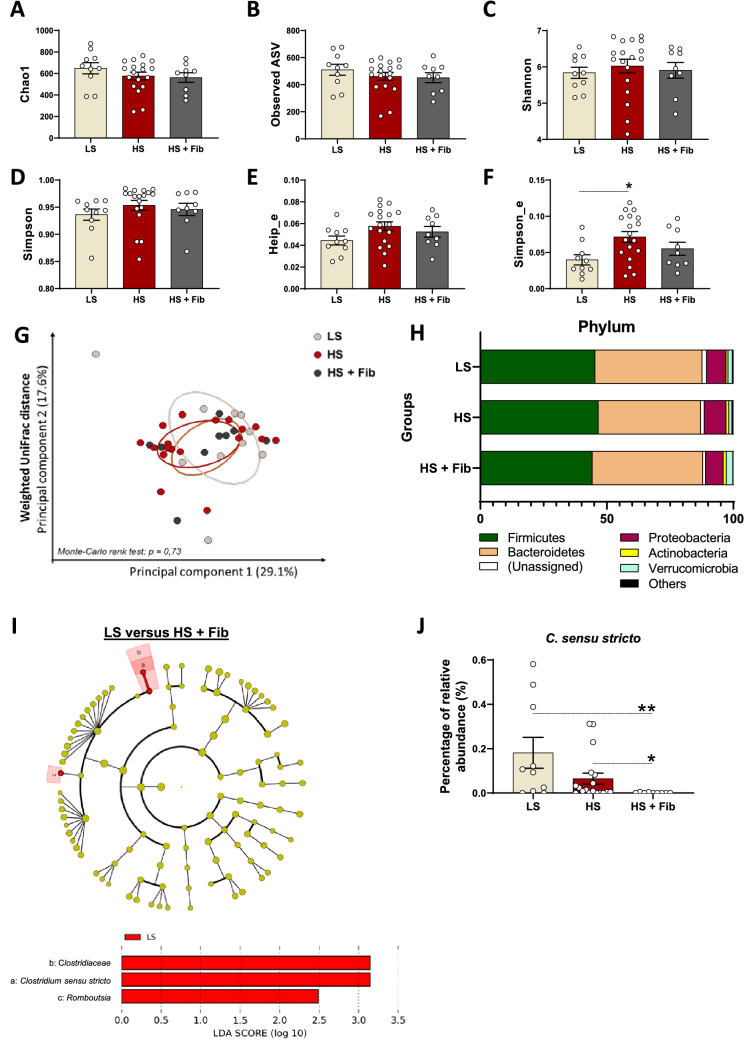
Overall gut microbiota composition in obese individuals with liver steatosis and/or liver fibrosis. Measure of alpha-diversity indexes: chao-1 (**A**), number of observed ASV (**B**), Shannon (**C**), Simpson (**D**), Heip-evenness (**E**) and Simpson-evenness (**F**), (n = 10 for patients with slight/moderate steatosis “LS”, n = 18 for patients with a high-severity of liver steatosis “HS” and n = 9 for patients with a HS and liver fibrosis “HS + Fib”). For each panel, data are expressed as mean ± SEM. A One-way ANOVA, followed by a Dunn’s multiple comparisons test, were performed. (**G**) Principal coordinates analysis (PCoA) of the weighted UniFrac distance, colored by group (PcoA was performed in R software version 3.5.1). (**H**) Barplots representing the mean of relative abundance of phyla, accounting for more than 1%, in each group. Graphical representations of alpha-diversity indexes and barplots were performed using Graphpad Prism software version 8 (www.graphpad.com). (**I**) Discriminant analysis of the fecal gut microbiota between LS and HS + Fib groups, using LefSe. Taxa enriched in the LS group are highlighted in red and shown in the linear discriminant analysis (LDA). Graphical representation was performed using Galaxy/Hutlab tool (huttenhower.sph.harvard.edu/galaxy). (**J**) Mean percentage of the relative abundance for *C. sensu stricto*. Data are expressed as mean ± SEM. A Kruskal–Wallis ANOVA test was performed for detecting significant differences between groups. Dunn’s multiple comparisons were then assessed. Significant differences are presented as: *p < 0.05, **p < 0.01.

Gut microbial changes related to the severity of liver steatosis and the presence of liver fibrosis was analyzed by comparing the gut microbiota of patients showing a slight/moderate steatosis (LS), with those exhibiting a high severity of liver steatosis (HS) and of patients with severe steatosis associated with liver fibrosis (HS + Fib). The α-diversity indices, related to bacterial richness (Chao1, Observed ASV), evenness (Heip evenness, Simpson evenness) or both (Shannon, Simpson), were measured. Globally, there is no major change in the gut bacterial diversity between groups (Fig. 2A–F). Only the Simpson evenness index significantly increased in patients with HS, compared to patients with LS (Fig. 2F). However, all indices related to bacterial richness were similar in all groups (Fig. 2A–D).

The overall gut microbiota composition was not different between the three groups as evidenced from a principal coordinate analysis of the weighted UniFrac distance (Fig. 2G). Moreover, no changes in the gut microbiota composition were observed at the phylum level between the three groups (Fig. 2H) and at the class and order levels (data not shown). At the family and genus level, the only significant change detected was a decrease of the *Clostridium sensu stricto* genus (Fig. 2J)-and its respective Clostridiaceae family-, especially in HS + Fib patients compared to LS (Table 5). Microbes discriminant for liver alterations were determined through a pairwise comparison using linear discriminant analysis effect size (LEfSe) (Fig. 2I and supp. Fig. 1A,B). For the LS-HS + Fib comparison, we still find that Clostridiaeceae and *Clostridium sensu stricto* but also *Romboutsia* are discriminant for the LS group (Fig. 2I). For the LS-HS comparison, *Flavonifractor* and *Faecalibacterium* are more represented in the HS group (Supp. Fig. 1A). Regarding the HS and HS + Fib comparison, we interestingly found that *Clostridium sensu stricto* characterized the HS group whereas *Escherichia/Shigella* is more represented in the gut microbiota from subjects with fibrosis (Supp. Fig. 1B).

**Table 5:** Taxa and ASV significantly altered with high-severity of liver steatosis and liver fibrosis in obese individuals.

Taxa		LS	HS	HS + Fib	*p* value	*p* value		
						(LS vs HS)	(LS vs HS + Fib)	(HS vs HS + Fib)
**Family**								
	Clostridiaceae_1	0.181 ± 0.069	0.065 ± 0.025	0.001 ± 0.001	**0.003**	0.172	**0.002**	**0.021**
***Genus***								
	*Clostridium_sensu_stricto*	0.181 ± 0.069	0.065 ± 0.025	0.001 ± 0.001	**0.003**	0.172	**0.002**	**0.021**
***ASV***								
*ASV_2148*	*Uncl. bacteria*	0.001 ± 0.001	0.014 ± 0.006	0.014 ± 0.004	**0.018**	**0.031**	**0.025**	0.459
*ASV_144*	*Uncl. Firmicutes*	0.177 ± 0.12	0.015 ± 0.012	0.008 ± 0.008	**0.008**	**0.012**	**0.017**	0.947
*ASV_346*	*Uncl. Firmicutes*	0.047 ± 0.02	0.012 ± 0.01	0.000 ± 0.000	**0.001**	**0.002**	**0.002**	0.504
*ASV_397*	*Uncl. Firmicutes*	0.012 ± 0.009	0.003 ± 0.002	0.000 ± 0.000	**0.039**	0.101	**0.039**	0.303
*ASV_664*	*Uncl. Clostridia*	0.043 ± 0.037	0.01 ± 0.005	0.051 ± 0.019	**0.014**	0.864	**0.030**	**0.016**
*ASV_2050*	*Uncl. Bacteroidales*	0.008 ± 0.004	0.065 ± 0.02	0.053 ± 0.019	**0.049**	0.076	0.057	0.857
*ASV_35*	*Uncl. Clostridiales*	1.12 ± 0.484	0.323 ± 0.093	0.154 ± 0.061	**0.013**	**0.029**	**0.015**	0.687
*ASV_560*	*Uncl. Lachnospiraceae*	0.026 ± 0.017	0.023 ± 0.014	0.000 ± 0.000	**0.026**	0.230	**0.022**	0.093
*ASV_592*	*Uncl Lachnospiraceae*	0.024 ± 0.015	0.041 ± 0.015	0.000 ± 0.000	**0.008**	0.786	**0.023**	**0.008**
*ASV_877*	*Uncl Lachnospiraceae*	0.001 ± 0.001	0.002 ± 0.001	0.025 ± 0.015	**0.019**	0.950	**0.026**	**0.027**
*ASV_248*	*Uncl. Ruminococcaceae*	0.102 ± 0.055	0.000 ± 0.000	0.035 ± 0.018	**0.012**	**0.021**	0.664	0.051
*ASV_439*	*Uncl. Ruminococcaceae*	0.02 ± 0.011	0.006 ± 0.004	0.116 ± 0.041	**0.003**	0.267	0.054	**0.002**
*ASV_370*	*Alistipes*	0.002 ± 0.002	0.052 ± 0.016	0.035 ± 0.032	**0.035**	0.052	0.640	0.114
*ASV_585*	*Romboutsia*	0.011 ± 0.003	0.02 ± 0.007	0.000 ± 0.000	**0.013**	0.974	**0.022**	**0.016**
*ASV_345*	*Roseburia*	0.109 ± 0.085	0.02 ± 0.016	0.001 ± 0.001	**0.047**	0.070	0.057	0.889
*ASV_422*	*Alistipes ihumii_99%*	0.059 ± 0.026	0.012 ± 0.009	0.010 ± 0.010	**0.047**	0.067	0.062	0.506
*ASV_561*	*Parabacteroides johnsonii_100%*	0.057 ± 0.025	0.01 ± 0.007	0.000 ± 0.000	**0.039**	0.179	**0.032**	0.174
*ASV_20*	*Sutterella wadsworthensis_100%*	0.291 ± 0.245	1.8 ± 0.429	0.041 ± 0.027	**0.001**	**0.015**	0.403	**0.002**
*ASV_75*	*Sutterella wadsworthensis_100%*	0.041 ± 0.028	0.095 ± 0.261	0.040 ± 0.027	**0.007**	**0.017**	0.871	**0.026**

Values refer to the mean percentage of relative abundance ± SEM**. LS:** Slight/moderate steatosis; **HS:** High-severity of steatosis; **HS + Fib:** High-severity of steatosis associated with fibrosis (n = 10 for LS, n = 18 for HS and n = 9 HS + Fib). Uncl.: unclassified. Kruskal–Wallis ANOVA test was performed for detecting significant differences between groups. Dunn’s multiple comparisons were then assessed. Significant differences are presented in bold.

**Figure 3:**
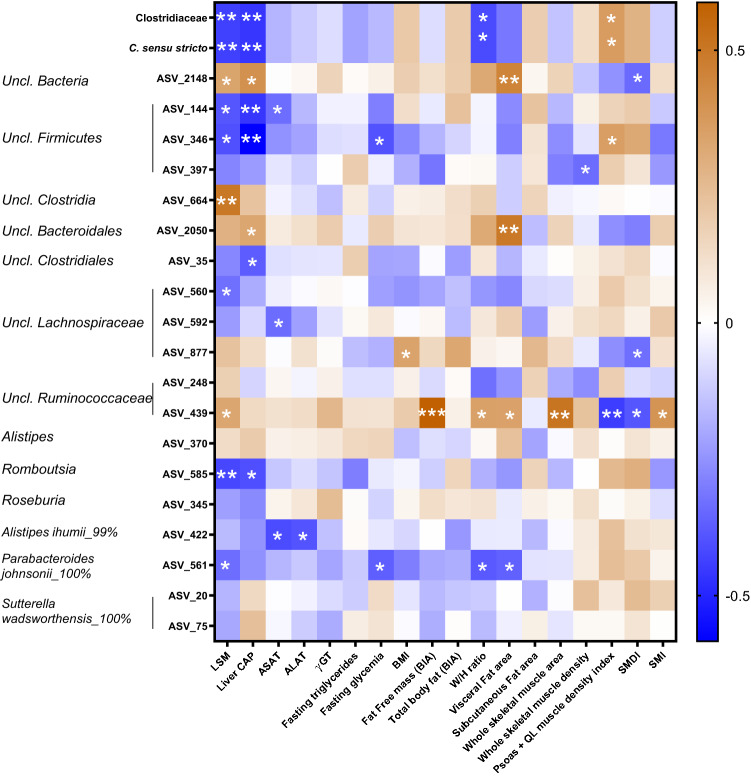
Correlations between taxa or ASV regulated in patients with high-severity of liver steatosis and/or liver fibrosis and metabolic features. Heatmap of Spearman’s correlations between taxa or ASV significantly different between LS, HS or HS + Fib patients and the most significant differences observed in metabolic outcomes. *p < 0.05, **p < 0.01 and ***p < 0.001 for significant correlations between parameters. Uncl., unclassified; LSM, liver stiffness measurement; CAP, controlled attenuation parameter; ASAT, aspartate aminotransferase; ALAT, alanine aminotransferase; γGT, γ-glutamyl transferase; BMI, body mass index; W/H ratio, waist to hip ratio; QL, quadratus lumborum muscle; SMDI, skeletal muscle density index; SMI, skeletal muscle index. Heatmap representation was performed using Graphpad Prism software version 8 (www.graphpad.com).

Amplicon sequence variants (ASV) analysis determined if specific sequences were different between the three groups. 19 ASV were differently expressed, according to liver steatosis and/or fibrosis (Table 5). Correlation analysis highlighted many associations between clinical parameters mentioned above and the bacterial changes observed in the gut microbiota of patients (Fig. 3). For the genera significantly changed, *Clostridium sensu stricto* negatively correlated with LSM, CAP and the waist to hip whereas it was positively associated with psoas and quadratus lumborum muscle density index. For ASV, we observed that ASV561 (belonging to the *Parabacteroides* genus) was decreased in HS and HS + Fib patients and negatively correlated with LSM, fasting glycemia, waist/hip ratio and visceral fat. ASV_422, showing 99% of similarity with *Alistipes ihumii,* was more expressed in the LS group and negatively correlated with liver enzymes ASAT and ALAT. In addition, one ASV belonging to *Romboutsia* genus (a discriminant genus when comparing LS and HS + Fib group using LEfSe) was less expressed in the HS + Fib group and was negatively associated with LSM and liver CAP. Interestingly, some other ASV, that cannot be classified at a genus level, increased in patients with liver fibrosis (ASV439: unclassified Ruminococcaceae) and positively correlated with fat-free mass, waist/hip ratio, LSM, visceral fat and whole muscle area, but negatively correlated with psoas and QL muscle density index. Some other unclassified ASV, belonging to Firmicutes phylum, are more represented in the LS group and negatively correlated with LSM and liver CAP (ASV_144, ASV_346).

## Discussion

The first objective of the dataset was to evaluate the relevance of TE to characterize the liver status of obese patients. We are conscious that one important limitation of our study is the absence of liver biopsy. Liver biopsy is still considered as the gold standard to validate steatosis and fibrosis stages, as well as the presence of hepatocyte ballooning and inflammation. However, liver biopsy has well-recognized drawbacks such as pain, anxiety and sampling variability^15^. Magnetic resonance imaging (MRI) based-techniques are the most non-invasive sensitive approaches for the detection of steatosis (MRI-proton density fat fraction) and fibrosis (magnetic resonance elastography)^21^. Anyway, this is a cost and time-consuming method, with limited availability. Some new modules of elastography are used in clinical practice with similar diagnostic accuracy for fibrosis^22^. However, they do not have the same validity and approved quality criteria as TE^3,17^ and do not provide any information on steatosis quantification. For all those reasons, we decided to perform TE. Indeed, this technique is suitable for routine use, performs better than ultrasound (widely used as the first-line assessment for MAFLD screening in nutritional studies^23–25^ and is the only technique able to provide at the same time a result on both steatosis and fibrosis evaluation.

Interestingly, we demonstrated that TE, indeed, gives reliable results in more than 90% of the patients, which is higher when compared with older studies reporting 5% of failures and ~ 15% of unreliable results^18^. Potential explanations are the use of first standard guidance ultrasound for the detection of the area of interest, the criteria used for reliability^19^ and finally a TE machine with both M and XL probes^18^.

In our cohort, all obese patients have steatosis, among which 73% had severe steatosis. However, the mean liver stiffness value was low, compatible with the absence of fibrosis in most patients, fibrosis being suspected in 27% of the patients. Although the cut-offs chosen for elasticity are high (7.8 kPa with M probe and 6.4 kPa with XL probe), the mean elasticity values remain low in our cohort and unrepresentative of a classical NASH patients cohort. In our study, severe steatosis and fibrosis more often affected men. Evidence from longitudinal studies suggest that the prevalence of MAFLD is indeed higher in the male as compared to the female gender, consistent with the notion that MAFLD is strongly linked with hormonal influences^26^. Regarding fibrosis risk, several studies also highlighted male sex^27^ and interestingly postmenopausal status^28^ as risk factors for MAFLD associated fibrosis.

MAFLD has to be interpreted as one part of a global metabolic situation. Interestingly, the recruitment of this study was done prospectively allowing a standard complete evaluation (blood analysis, TE, BIA, CT-scan and stool sample). CT images provide a precise information on specific adipose tissue and muscles, not available with the BIA^29^. Our results showed that severe steatosis correlates with ALAT values and was associated with visceral adiposity and insulin resistance (high blood glucose level). In contrast, liver fibrosis correlated better with ASAT values. Interestingly, our study highlighted an association between body composition and liver status. Indeed, we already know that abdominal adiposity plays a pathogenic role in MAFLD^29^. With our study, we were able to determine that visceral adipose tissue expansion was a key characteristic of severe steatosis. Furthermore, we showed that muscle alterations were a strong feature of both steatosis and development of fibrosis, highlighting. the importance of skeletal muscle evaluation in the context of MAFLD^30^. In patients with MAFLD, a higher SMI (muscle area adjusted for height) was observed in the context of severe steatosis. This situation (at early time points of MAFLD without fibrosis) is in contrast with the lower muscle mass (sarcopenia) evidenced in late end stage liver diseases^9^. We show that patients with fibrosis have higher fat free mass (at BIA), whole muscle area (at CT) and a lower density index (SMDI) that reveals myosteatosis^31^. Hence, patients with increased steatosis severity have increased muscle mass, and those with fibrosis have decreased muscle quality (i.e. higher degree of fatty infiltration) and this association persisted when only selected muscle group were considered (*quadratus lomborum* and *psoas*). The translation of this finding in terms of muscle functionality remains to be established. Indeed, a lower grip strength has been previously described to be associated with MAFLD^32–34^. Also, whether muscles changes are mere consequences of the dysmetabolic status of these patients or whether they drive liver fibrosis progression remains unknown. Of note, the increased muscle mass seen in patients with severe steatosis compared to patients with non-severe steatosis contrasts with recent data on MAFLD patients, wherein low muscle mass is associated with the severity of steatosis and fibrosis ^35^. However, as already shown in the past^36^ and recently highlighted^23^, MAFLD is only associated with sarcopenia when using the weight (or BMI)-adjusted SMI. In contrast, it showed the opposite result when using the height-adjusted SMI^23^. In our study, this issue is leveled out by the use of CT-SMI, as this gold standard index is scaled on height thus not directly influenced by obesity. The results on the association of myosteatosis and liver fibrosis also contrasts with recent data on MAFLD in which the authors do not find a link between the muscle fat fraction measured with chemical shift gradient echo MRI and liver stiffness^37^. However, the muscle fat fraction was calculated in one region of interest without any adjustment to the muscular surface^37^. By the way, we show similar results, i.e. a similar muscle density between the two groups but we provide one additional element, i.e. a significantly decreased muscle density index in patients with fibrosis compared to the others, reflecting increased absolute myosteatosis in patients with fibrosis.

Finally, the literature clearly demonstrated that the gut microbiota could be an important component to consider in MAFLD^13^. We therefore wanted to associate the extra-hepatic elements studied above (anthropometric data, adiposity, muscular compartment, biological profile) with the evaluation of the gut microbiota. FLORINASH study revealed that obese patients with steatosis had low microbial gene richness^10^. Another study showed that the abundance of some bacterial species belonging to *Alistipes*, *Bacteroides, Clostridium, Coprococcus, Eubacterium, Ruminococcus* or *Roseburia* genera (among others) were different in the gut microbiota of MAFLD patients with advanced fibrosis compared to moderate fibrosis^38^. In our study, we showed that the severity of steatosis and fibrosis in obese patients was related to very minor changes in microbial diversity -only one evenness index was associated with the severity of steatosis-, whereas we also pointed out a regulation of two ASV belonging to *Alistipes* and one belonging to *Roseburia*. Interestingly, we identified *Clostridium sensu stricto* as the only genus decreasing with the development of fibrosis. A previous study reported a decrease of *Clostridium sensu stricto* in MAFLD patients versus healthy controls^39^. Here, we reported that this genus is the only one regulated with the progression of the disease, since it decreased in patients with severe steatosis associated with fibrosis, compared to patients with moderate or severe steatosis without fibrosis. In addition, this genus negatively correlated with both LSM and CAP measurements and with the waist to hip ratio. Interestingly, *Clostridium sensu stricto* was positively associated with the muscle density index of *psoas* and *quadratus lomborum*. The reduced muscle density was identified in our study as a strong factor correlated with fibrosis development, thus identification of some bacteria associated with both factors is of relevance. We also observed that an ASV belonging to *Alistipes* genus (ASV_422) decreased with the severity of steatosis and negatively correlated with liver enzymes, whereas another ASV for *Alistipes* (ASV_370) increased with severe steatosis but was not associated to any clinical outcomes assessed in tour study. The *Alistipes* genus was lower in both children and adult MAFLD patients versus healthy individuals^40,41^. In addition, the ASV_585 belonging to *Romboutsia* genus decreased in the feces of patients exhibiting liver fibrosis and the linear discriminant analysis (LDA) revealed that the *Romboutsia* genus was more present in the feces of LS group, when compared with HS + Fib group. Interestingly, the *Romboutisa* ASV_585 negatively correlated with both LSM and CAP measurements. We found only one reference linking the *Romboutsia* genus with liver complications in mice^42^. In this study, a glucagon-like peptide-1 analog (liraglutide) improved fatty liver in *db/db* mice and this was associated with an enrichment of *Romboutsia* genus in the colon content. In this rodent model, *Romboutsia* genus negatively correlated with the serum activity of liver enzymes ASAT and ALAT, supporting our observations that this genus is associated with liver alterations. The relative abundance of another ASV belonging to *Parabacteroides* genus (ASV_561) decreased with in HS + Fib group, compared to HS group, and negatively correlated with LSM measurement, fasting glycemia, visceral fat and waist/hip ratio. In the literature, it has been already shown that the abundance of *Parabacteroides* tended to be lower in pediatric MAFLD patients compared to control subjects, suggesting that the presence of this genus can be also interesting in the context of liver complications^43^. Finally, comparisons between HS and HS + Fib groups using LEfSe analysis also highlighted that Enterobacteriaceae and *Escherichia/Shigella* were enriched in the group of patients with liver fibrosis. In NASH children, compared to obese or healthy children, an increase of both Enterobacteriaceae and *Escherichia* genus were also previously found^41^. In our cohort of obese adults, it seems that these regulations were observed with liver fibrosis and confirm previous observations showing that Enterobacteriaceae and *Escherichia/Shigella* were enriched in patients with significant fibrosis, compared to patients F0/F1 fibrosis^44^.

**Figure 4:**
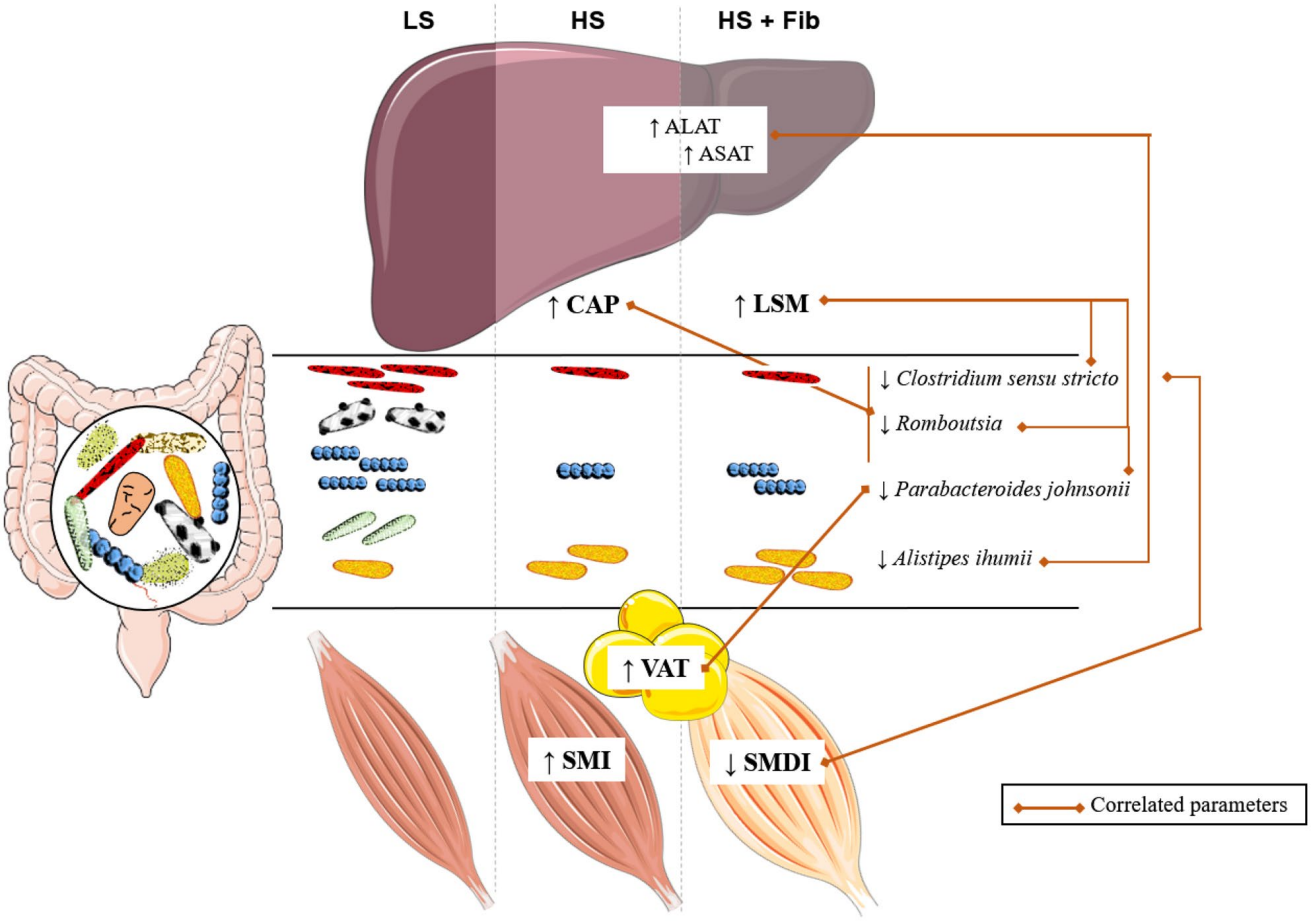
Muscle dysfunction, specific gut microbiota characteristics, and visceral adiposity correlate to the severity of metabolic associated fatty liver disorders (MAFLD) in obese patients. CAP, controlled attenuation parameter; LS, low degree of steatosis; HS, high degree of steatosis; HS + Fib, high degree of steatosis and fibrosis; LSM, liver stiffness measurement; SMI, skeletal muscle index; SMDI, skeletal muscle density index; VAT, visceral adipose tissue. The figure was produced using Servier Medical Art (www.servier.com).

In conclusion, fibrosis could be easily detected in obese patients by TE which measures simultaneously both liver stiffness and the controlled attenuation parameter. Our data lead us to identify a set of extrahepatic characteristics distinguishing fibrosis development and steatosis severity estimated by TE. Increased ALAT values, expansion or visceral adipose tissue and increased muscle mass are good indicators of steatosis grade, while high ASAT values, and a decrease in muscle density index are associated with fibrosis development. Further studies are needed to validate these data in relation to liver histology, in particular in patients with biopsy-proven liver fibrosis, and to evaluate whether muscle myosteatosis actively acts on liver disease or could simply be the consequence of advanced metabolic/liver situation. Among gut microbial taxa, *Clostridium sensu stricto* decreased with the development of liver fibrosis and related muscle density. Taken together, the data from our study highlight an extra-hepatic signature (myosteatosis and reduction of *Clostridium sensu stricto*) associated with the appearance of fibrosis, assessed by TE (Fig. 4). In patients with severe steatosis, the increase of *Escherichia/Shigella* seems to be discriminant for the presence of liver fibrosis. Other bacterial ASV were pointed out as potential markers to be evaluated in larger cohorts to unravel their link with the degree of MAFLD severity, such as ASV belonging to *Romboutsia, Alistipes* or *Parabacteroides,* as well as their potential involvement in the production of microbial metabolites able to modulate liver dysfunction.

## Methods

### Subjects

Subjects were recruited at the outpatient clinic of Cliniques universitaires Saint-Luc for a 3-month multicenter interventional trial^20^. We evaluated baseline data, i.e. before intervention, of patients, in order to have all the data analyzed by a TE machine equipped with the M and XL probes as well as with the CAP technique. The inclusion criteria were obesity (BMI ≥ 30 kg/m^2^), age (18–65 years), Caucasian ethnicity and presence of at least one metabolic feature: prediabetes, diabetes, dyslipidemia, hypertension, elevated alanine aminotransferase (ALAT) or aspartate aminotransferase (ASAT) or γ-glutamyl transferase (γGT) suggestive of MAFLD. Exclusion criteria were type 1 diabetes, significant alcohol consumption, other cause of chronic liver disease (e.g. viral hepatitis, genetic disease), recent (< 6 weeks) antibiotic use or special dietary requirement or supplement. This study was approved by the “Comité d’éthique hospitalo-facultaire de Saint-Luc”. The trial protocol was published on protocols.io (https://doi.org/10.17504/protocols.io.baidica6) and the trial was registered at ClinicalTrials.gov (NCT03852069; 22/09/2019). The authors ensure that the study has been carried out in accordance with The Code of Ethics of the World Medical Association and followed the ethical guidelines set out in the Declaration of Helsinki. All participants provided written informed consent in compliance with the European law 2001/20/CE guidelines.

### Procedure

Weight, height, waist and hip circumferences and blood pressure were measured. The use of lipid-lowering, anti-hypertensive and diabetes medications was recorded. Liver stiffness and CAP measurements were performed using FIBROSCAN (Echosens, Paris, France) by one single experienced operator in clinical routine and research. The patients were lying in a dorsal position with the right arm in abduction. Measurements were performed in the right lobe of the liver, through one intercostal space, chosen under standard ultrasound guidance. The M or XL-probe was chosen based on the automatic recommendation of the device. All measurements were performed in a fasted (overnight) condition. Results were expressed as the median and the interquartile range (IQR). Failure of LSM was defined as the absence of valid measurement. Reliability of LSM was defined by previously established criteria^19^. A total of 15 valid measurements with a LSM success rate > 70% and an interquartile range/median < 30% for values > 7.0 kPa^19^ were considered as reliable results. Patients were then stratified based on the result of the CAP and liver stiffness measurements. LSM ≥ 7.8 kPa with the M probe or ≥ 6.4 with the XL probe were defined as advanced fibrosis stages, as recommended in the literature^18^ and used in our daily center clinical practice^45^. For steatosis in the context of MAFLD, the following cut-offs were used: 215 dB/m for slight steatosis (grade S1), 252 dB/m for moderate steatosis (grade S2) and 296 dB/m for severe steatosis (grade S3) as already described in the literature^16^ and used in our clinical practice^45^.

Glycaemia, HbA1c, ASAT, ALAT, γGT and triglycerides were measured in fasting plasma samples.

Body composition was assessed by bio-impedance devices (BIA 101, Akern, Italy; Biocorpus, Medi Cal, Germany; Tanita BC-418 MA, Tanita, UK). The measure of resistance obtained was used to calculate total body fat and fat free mass (expressed in kg).

Body composition was also measured from the abdominal CT scan at the third lumbar (L3) level (performed on the same day as TE) with Slice-O-Matic software, version 4.3 (Tomovision, Montreal, Canada) using well previously described methodology^46^. Briefly, subcutaneous fat area, visceral fat area and skeletal muscle area and density were delineated using Hounsfield Unit (HU) values at the commonly accepted threshold of − 190 to – 30 HU; − 150 to − 50 HU and − 29 to + 150 HU, respectively^46^. Whole skeletal muscle area was normalized for stature and was referred to as the skeletal muscle index (SMI) (cm^2^/m^2^). Mean muscle density, a proxy for muscle fat content^31^, was measured. We then normalized skeletal muscle density to skeletal muscle area to reflect the absolute amount of fat per muscle and this ratio is referred to as the whole skeletal muscle density index (SMDI) (HU/cm^2^). All CT images were analyzed by two trained observers (PT and MN), blinded for liver steatosis and stiffness evaluation.

### DNA extraction and 16S rRNA gene sequencing

Stool samples were available for 37 patients (n = 10 for patients with non-severe liver steatosis, n = 18 for patients with severe liver steatosis and n = 9 for patients with both severe steatosis and fibrosis). Stool samples were stored at room temperature with a DNA stabilizer (Stratec biomolecular, Berlin, Germany) for maximum three days, then transferred to − 80 °C for the analysis of the gut microbiota composition. Genomic DNA was extracted from feces using a PSP spin stool DNA kit (Stratec biomolecular, Berlin, Germany) 0.16SrRNA gene sequencing and subsequent bioinformatics and biostatistics analyses were performed as previously described^20^ and detailed in Supplementary File 1.

### Statistical analysis

Data are expressed as mean ± SD. For the comparison between baseline characteristics, Student t-test was used for continuous variable and chi-squared test was used for categorical variables. A significance level of p < 0.05 was used for all the analyses.

Multivariate analyses were performed on SPSS (v24) using binary logistic regression. All parameters were systematically checked for collinearity.

For gut microbiota analysis, data are expressed as the mean percentage of relative abundance ± SEM. A Kruskal–Wallis ANOVA test was performed for detecting significant differences for taxa and ASV between groups, followed by a Dunn’s multiple comparisons test, using R software (version 3.5.1) and the FSA package. A significance level of p < 0.05 was used for identifying taxa and ASV that are significantly expressed between groups. Taxa with a mean percentage of relative abundance below 0.01% were removed. To avoid analyzing spurious sequences, the ASVs for which the overall mean of relative abundance is below 0.01%, and detectable in less than 20% of samples, were removed. P-values were then corrected to control for the false discovery rate (FDR) for multiple tests according to the Benjamini and Hochberg procedure and a significance level of q < 0.05 was used. Beta-diversity clustering was analyzed using a Monte-Carlo rank test. Discriminant taxa were also identified using LEfSe^47^. LEfSe algorithm measures statistical significance using the Kruskal–Wallis test and biological consistency using the Wilcoxon rank sum test. Associations between the taxa and ASV differently expressed between groups and the clinical outcomes were assessed by Spearman’s correlation tests. A significance level of *p* < 0.05 was also adopted and the Heatmap of correlation was visualized with the GraphPad Prism 8 software.

## Supplementary Information

Supplementary Information.

## Abbreviations

ALAT: Alanine aminotransferase
ASAT: Aspartate aminotransferase
BIA: Bioimpedance analysis
BMI: Body mass index
CAP: Controlled attenuation parameter
CT: Computed tomography
γGT: γ-glutamyl transferase
LSM: Liver stiffness measurement
MAFLD: Metabolic dysfunction-associated fatty liver disease
SMDI: Skeletal muscle density index
SMI: Skeletal muscle index
TE: Transient elastography
