# Publisher Correction: Microbiota analysis and transient elastography reveal new extra-hepatic components of liver steatosis and fibrosis in obese patients

**DOI:** 10.1038/s41598-021-85083-y

**Published:** 2021-03-11

**Authors:** Nicolas Lanthier, Julie Rodriguez, Maxime Nachit, Sophie Hiel, Pierre Trefois, Audrey M. Neyrinck, Patrice D. Cani, Laure B. Bindels, Jean-Paul Thissen, Nathalie M. Delzenne

**Affiliations:** 1 Laboratory of Hepatology and Gastroenterology, Institut de Recherche Expérimentale et Clinique, UCLouvain, Université catholique de Louvain, Brussels, Belgium; 2 Service d’Hépato-gastroentérologie, Cliniques universitaires Saint-Luc, UCLouvain, Brussels, Belgium; 3 Metabolism and Nutrition Research Group, Louvain Drug Research Institute, UCLouvain, Université catholique de Louvain, Avenue E. Mounier, box B1.73.11, 1200 Brussels, Belgium; 4 Medical and Imaging Department, Cliniques universitaires Saint-Luc, UCLouvain, Brussels, Belgium; 5 WELBIO - Walloon Excellence in Life Sciences and BIOtechnology, UCLouvain, Université catholique de Louvain, Brussels, Belgium; 6 Pole of Endocrinology, Diabetes and Nutrition, Institut de Recherche Expérimentale et Clinique, UCLouvain, Université catholique de Louvain, Brussels, Belgium; 7 Service d’Endocrinologie, diabétologie et nutrition, Cliniques universitaires Saint-Luc, UCLouvain, Brussels, Belgium

Correction to: *Scientific Reports* https://doi.org/10.1038/s41598-020-79718-9, published online 12 January 2021

The original version of this Article contained errors.

Patrice D. Cani was incorrectly affiliated with ‘Medical and Imaging Department, Cliniques universitaires Saint-Luc, UCLouvain, Brussels, Belgium’. The correct affiliations for Patrice D. Cani are listed below:

Metabolism and Nutrition Research Group, Louvain Drug Research Institute, UCLouvain, Université catholique de Louvain, Avenue E. Mounier, box B1.73.11, 1200 Brussels, Belgium.

WELBIO - Walloon Excellence in Life Sciences and BIOtechnology, UCLouvain, Université catholique de Louvain, Brussels, Belgium

In addition, the original version of this Article contained a repeated error where,

*“Clostridium* sensu stricto”

now reads:

*“Clostridium* *sensu stricto*”

These errors have now been corrected in the PDF and HTML versions of the Article.

