# Supplementary material for: Microbiota analysis and transient elastography reveal new extra-hepatic components of liver steatosis and fibrosis in obese patients
Source: Sci Rep. 2021 Jan 12;11:659. doi: 10.1038/s41598-020-79718-9 (PMC7804131; doi:10.1038/s41598-020-79718-9)
Supplement: Supplementary file 1 — Supplementary Information. [file 41598_2020_79718_MOESM1_ESM.docx]

**Supplementary File 1**

**Title:** Microbiota analysis and transient elastography reveal new extra-hepatic components of liver steatosis and fibrosis in obese patients

**Authors :** Nicolas Lanthier, Julie Rodriguez, Maxime Nachit, Sophie Hiel, Pierre Trefois, Audrey M. Neyrinck, Patrice D. Cani, Laure B. Bindels, Jean-Paul Thissen, Nathalie M. Delzenne

DNA extraction and 16S rRNA gene sequencing

Stool samples were available for 37 patients (n=10 for patients with non-severe liver steatosis, n=18 for patients with severe liver steatosis and n=9 for patients with both severe steatosis and fibrosis). Stool samples were stored at room temperature with a DNA stabilizer (Stratec biomolecular, Berlin, Germany) for maximum three days, then transferred to -80°C for the analysis of the gut microbiota composition. Genomic DNA was extracted from feces using a PSP^®^ spin stool DNA kit (Stratec biomolecular, Berlin, Germany).

Amplicon sequencing of the microbiome was done at the University of Minnesota Genomics Center. Briefly, the V5-V6 region of the 16S rRNA gene was PCR-enriched using the primer pair V5F_Nextera (TCGTCGGCAGCGTCAGATGTGTATAAGAGACAG**RGGATTAGAT**

**ACCC**) and V6R_Nextera (GTCTCGTGGGCTCGGAGATGTGTATAAGAGACAG**CGAC**

**RRCCATGCANCACCT**) (in bold, primers 784F and 1064R, amplicon size of 280 bp) in a 25 μl PCR reaction containing 5 μl of template DNA, 5 μl of 2X HotStar PCR master mix, 500 nM of final concentration of primers and 0.025 U/μl of HostStar Taq+ polymerase (QIAGEN). PCR-enrichment reactions were conducted as follows : an initial denaturation step at 95°C for 5 min followed by 25 cycles of denaturation (20 s at 98°C), annealing (15 s at 55°C), and elongation (1 min at 72°C), and a final elongation step (5 min at 72°C). Next, the PCR-enriched samples were diluted 1:100 in water for input into library tailing PCR. The PCR reaction was analogous to the one conducted for enrichment except with a KAPA HiFi Hot Start Polymerase concentration of 0.25 U/ μl, while the cycling conditions used were as follows, initial denaturation at 95°C for 5 min followed by 10 cycles of denaturation (20 s at 98°C), annealing (15 s at 55°C), and elongation (1 min at 72°C), and a final elongation step (5 min at 72°C). The primers used for tailing are the following: F-indexing primer AATGATACGGCGACCACCGAGATCTACAC[i5]TCGTCGGCAGCGTC and R-indexing primer CAAGCAGAAGACGGCATACGAGAT[i7]GTCTCGTGG GCTCGG, where [i5] and [i7] refer to the index sequence codes used by Illumina. The resulting 10μl indexing PCR reactions were normalized using a SequalPrep normalization plate according to the manufacturer’s instructions (Life Technologies). 20 μl of each normalized sample was pooled into a trough, and a SpeedVac was used to concentrate the sample pool down to 100 μl. The pool was then cleaned using 1X AMPureXP beads and eluted in 25 μl of nuclease-free water. The final pool was quantitated by QUBIT (Life Technologies) and checked on a Bioanalyzer High-Sensitivity DNA Chip (Agilent Technologies) to ensure correct amplicon size. The final pool was then normalized to 2 nM, denatured with NaOH, diluted to 8 pM in Illumina’s HT1 buffer, spiked with 20% PhiX, and heat denatured at 96°C for 2 minutes immediately prior to loading. A MiSeq 600 cycle v3 kit (2x300) was used to sequence the pool.

Subsequent bioinformatics and biostatistics analyses were performed *in house* in a Linux environment. Initial quality filtering of the reads was performed with the Illumina Software, yielding an average of 89238 pass-filter clusters per sample. Quality scores were visualized with the FastQC software v0.10.1 (http://www.bioinformatics.babraham.ac.uk/ publications.html), and reads were trimmed to 220 bp (R1) and 200 bp (R2) with the FASTX-Toolkit 0.013 (http://hannonlab.cshl.edu/fastx_toolkit/). Next, reads were merged with the merge-illumina-pairs application v1.4.2 (with P = 0.03, enforced Q30 check, perfect matching to primers which are removed by the software, and otherwise default settings including no ambiguous nucleotides allowed) ^1^. For all the samples, a subset of 25000 reads (except for one sample in the LS group: 23562 reads) was randomly selected using Mothur v1.25.0 ^2^ to avoid large disparities in the number of sequences. Indeed, some diversity indexes are sensitive to sequencing depth ^3^. Subsequently, the USEARCH pipeline (v11.0.667) was used to further process the sequences ^4^. Amplicon sequence variants (ASVs) were identified using UNOISE3 ^5^. The analysis allowed the identification of 3305 ASVs. Taxonomic prediction was performed using the RDP database V16 and the *nbc_tax* function ^6^, an implementation of the RDP Naive Bayesian Classifier algorithm ^7^. Taxonomy for significant ASV was also confirmed using the EZ BioCloud 16S database (update 2020.10.12) and the JGI IMG platform (Integrated Microbial Genomes and Metagenomes biocomputational system developed from the Joint Genome Institute). The phylotypes were computed as percent proportions based on the total number of sequences in each sample. Alpha diversity indexes and beta diversity indexes were calculated using QIIME 1.8.0 ^8^. PcoA plot of the beta-diversity indexes were visualized using R software (version 3.5.1), using the ade4 package. Barplot for phylum was visualized on the GraphPad Prism version 8.0 software.

Raw sequences can be accessed in SRA database (accession PRJNA595949).

**References**

1 Eren, A. M., Vineis, J. H., Morrison, H. G. & Sogin, M. L. A filtering method to generate high quality short reads using illumina paired-end technology. *PLoS One* **8**, e66643, doi:10.1371/journal.pone.0066643 (2013).

2 Schloss, P. D. *et al.* Introducing mothur: open-source, platform-independent, community-supported software for describing and comparing microbial communities. *Appl Environ Microbiol* **75**, 7537-7541, doi:10.1128/AEM.01541-09 (2009).

3 Reese, A. T. & Dunn, R. R. Drivers of Microbiome Biodiversity: A Review of General Rules, Feces, and Ignorance. *mBio* **9**, doi:10.1128/mBio.01294-18 (2018).

4 Edgar, R. C. UPARSE: highly accurate OTU sequences from microbial amplicon reads. *Nat Methods* **10**, 996-998, doi:10.1038/nmeth.2604 (2013).

5 Edgar, R. C. UNOISE2: improved error-correction for Illumina 16S and ITS amplicon sequencing. *bioRxiv preprint doi:* [*https://doi.org/10.1101/081257*](https://doi.org/10.1101/081257)*.* (2016).

6 Edgar, R. C. Accuracy of taxonomy prediction for 16S rRNA and fungal ITS sequences. *PeerJ* **6**, e4652, doi:10.7717/peerj.4652 (2018).

7 Wang, Q., Garrity, G. M., Tiedje, J. M. & Cole, J. R. Naive Bayesian classifier for rapid assignment of rRNA sequences into the new bacterial taxonomy. *Appl Environ Microbiol* **73**, 5261-5267, doi:10.1128/AEM.00062-07 (2007).

8 Caporaso, J. G. *et al.* QIIME allows analysis of high-throughput community sequencing data. *Nat Methods* **7**, 335-336, doi:10.1038/nmeth.f.303 (2010).


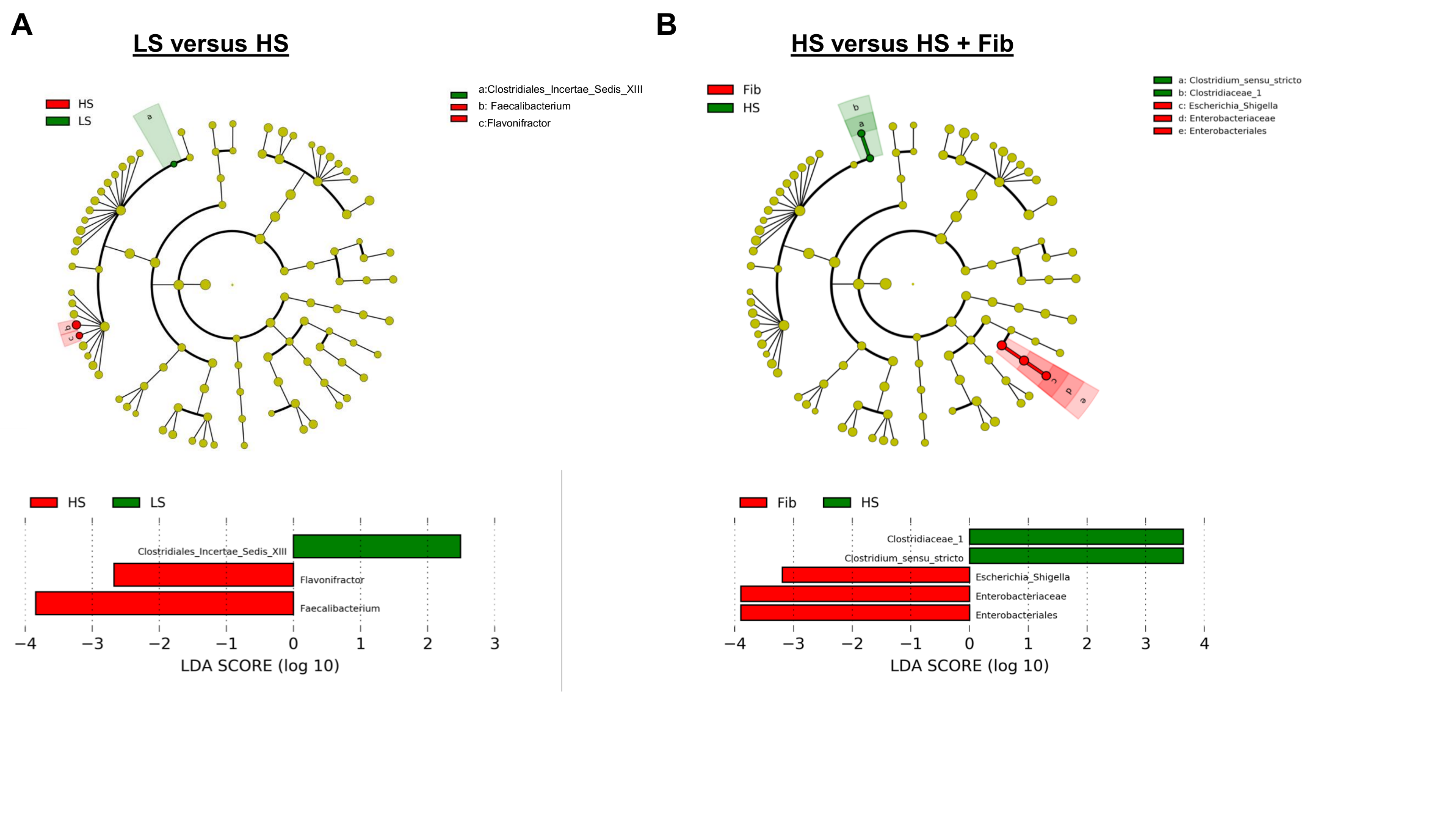


**Supplementary Figure S1.** Discriminant analysis of the fecal gut microbiota using LefSe. (a) Comparisons between LS and HS groups. Cladogram and linear discriminant analysis (LDA) score represents the taxa enriched in the LS group in green and those enriched in HS group in red. (b) Comparisons between HS and HS+Fib groups. Cladogram and linear discriminant analysis (LDA) score represent the taxa enriched in the HS group in green and those enriched in HS+Fib group in red. Graphical representation was performed using Galaxy/Hutlab tool (huttenhower.sph.harvard.edu/galaxy).
